# Sustained Isometric Wrist Flexion and Extension Maximal Voluntary Contractions Similarly Impair Hand-Tracking Accuracy in Young Adults Using a Wrist Robot

**DOI:** 10.3389/fspor.2020.00053

**Published:** 2020-05-08

**Authors:** Davis A. Forman, Garrick N. Forman, Maddalena Mugnosso, Jacopo Zenzeri, Bernadette Murphy, Michael W. R. Holmes

**Affiliations:** ^1^Faculty of Science, Ontario Tech University, Oshawa, ON, Canada; ^2^Faculty of Applied Health Sciences, Brock University, St. Catharines, ON, Canada; ^3^Robotics, Brain and Cognitive Sciences, Istituto Italiano di Tecnologia, Genoa, Italy; ^4^Faculty of Health Sciences, Ontario Tech University, Oshawa, ON, Canada

**Keywords:** forearm, performance fatigability, tracking, isometric, robotics, kinematics, flexion, extension

## Abstract

Due to their stabilizing role, the wrist extensor muscles demonstrate an earlier onset of performance fatigability and may impair movement accuracy more than the wrist flexors. However, minimal fatigue research has been conducted at the wrist. Thus, the purpose of this study was to examine how sustained isometric contractions of the wrist extensors/flexors influence hand-tracking accuracy. While gripping the handle of a three-degrees-of-freedom wrist manipulandum, 12 male participants tracked a 2:3 Lissajous curve (±32° wrist flexion/extension; ±18° radial/ulnar deviation). A blue, circular target moved about the trajectory and participants tracked the target with a yellow circle (corresponding to the handle's position). Five baseline tracking trials were performed prior to the fatiguing task. Participants then exerted either maximal wrist extension or flexion force (performed on separate days) against a force transducer until they were unable to maintain 25% of their pre-fatigue maximal voluntary contraction (MVC). Participants then performed 7 tracking trials from immediately post-fatigue to 10 min after. Performance fatigability was assessed using various metrics to account for errors in position-tracking, error tendencies, and movement smoothness. While there were no differences in tracking error between flexion/extension sessions, tracking error significantly increased immediately post-fatigue (Baseline: 1.40 ± 0.54°, Post-fatigue: 2.02 ± 0.51°, *P* < 0.05). However, error rapidly recovered, with no differences in error from baseline after 1-min post-fatigue. These findings demonstrate that sustained isometric extension/flexion contractions similarly impair tracking accuracy of the hand. This work serves as an important step to future research into workplace health and preventing injuries of the distal upper-limb.

## Introduction

Work by Holmes et al. (2015) demonstrated that the wrist extensor muscles of the forearm contribute more to joint rotational stiffness (JRS) than the wrist flexors during external wrist perturbations. As greater JRS tends to result in a greater resistance to sudden disturbances (Cholewicki and McGill, 1996; Brown and Potvin, 2007), the wrist extensor muscles have been labeled as the primary stabilizers of the wrist. Further evidence for this hypothesis comes from studies assessing muscle activity, or from studies calculating co-contraction from muscle activity, both of which are surrogate measures for joint stiffness (De Serres and Milner, 1991; Cholewicki and McGill, 1996; van Loon et al., 2001; Franklin and Milner, 2003). The wrist extensors exhibit significantly greater co-contraction during both handgrip forces and wrist exertions than the flexors (Forman et al., 2019). The wrist extensors also demonstrate less task-dependency; they exhibit high levels of activity regardless of task-parameters (Mogk and Keir, 2003; Forman et al., 2019). This continuous, elevated activity predisposes the wrist extensors to an earlier onset of fatigue (Hägg and Milerad, 1997) and is likely the primary reason why they demonstrate a higher incidence of overuse injuries than the flexors (Shiri et al., 2006). For instance, the prevalence of lateral epicondylitis (which affects the wrist extensors) is ~1–3% in the average population (Allander, 1974; Verhaar, 1994; Shiri et al., 2006, 2007), but can vary wildly in different occupational settings. In tennis players, the prevalence is thought to be closer to 35–40%, although this number seems to increase with age (Gruchow and Pelletier, 1979; Carroll, 1981). In mild cases, lateral epicondylitis can be treated with improved rest, physical therapy, and custom braces, but in severe cases, can result in prolonged work absence and require invasive surgery. Both the prevalence and the severity of lateral epicondylitis are worse than medial epicondylitis (affects the wrist flexors; approximate prevalence of 0.4% in the general population; Shiri et al., 2006).

Given the broad scope of fatigue as a field of study, and the inconsistency in which fatigue is defined, recent literature has proposed a taxonomy to provide clearer communication between studies (Kluger et al., 2013; Enoka and Duchateau, 2016). According to this work, fatigue should be defined as a symptom in which both physical and cognitive function may be limited through interactions of perceived fatigability and performance fatigability (Enoka and Duchateau, 2016). Perceived fatigability refers to the subjective state of the individual and thus involves subjective measures, while performance fatigability is measured through objective laboratory-based assessments characterizing the functional decline of performance (Marrelli et al., 2018). Performance fatigability (modulated by both muscle contractile function and by voluntary muscle activation, or classically termed peripheral and central fatigue; Kluger et al., 2013), can manifest experimentally as decreased movement accuracy (Missenard et al., 2008b), impaired proprioception acuity (Pedersen et al., 1999; Mugnosso et al., 2019), decreased co-contraction during precision movements (Gribble et al., 2003; Missenard et al., 2008b), and decreased peak contractile speed and torque generation (de Haan et al., 1989). These factors not only compromise joint stability but also contribute to greater signal-dependent noise (SDN; signal meaning the optimal, ideal force required to perform a task, and noise meaning any deviation from that ideal) (Missenard et al., 2008a). The result is an overall increase in force variability, which reduces the accuracy of precision movements. Greater movement error has real-world implications. While the consequences of performance fatigability can contribute to the development of chronic overuse injuries, impairments to movement accuracy may lead to performance decrements and greater risks of suffering acute injuries (Parijat and Lockhart, 2008). Understanding how performance fatigability manifests, and the specific ways that it impairs performance, is an important step in mitigating its potentially harmful effects. This is particularly important in the context of the distal upper-limb, as the hand makes the final interface with the external environment.

However, there is currently limited research into how performance fatigability develops in the forearm, with most work centered around office mouse use (Huysmans et al., 2008). Additionally, we are aware of only one study that has examined performance fatigability between opposing muscle groups (Jaric et al., 1997). In this study, agonist muscle fatigue caused greater velocity, acceleration, and deceleration deficits in the agonist than the antagonist (minimal differences were seen whether the agonists were the elbow flexors or extensors). There is insufficient literature to conclude what influence performance fatigability of the forearm has on hand-tracking accuracy. The potential specificity of performance fatigability between forearm muscle groups is also unknown. Therefore, the purpose of the present study was to examine hand-tracking accuracy prior to and following a single bout of maximal, sustained, isometric wrist extension, or wrist flexion contraction. Hand-tracking error was examined while performing on a three-degrees-of-freedom wrist manipulandum, and hand movement incorporated wrist flexion/extension and radial/ulnar deviation. Tracking error was expected to increase following sustained contractions of either muscle group. However, it was hypothesized that tracking error would be greater following wrist extension fatigue, given the evidence that the wrist extensors contribute more to wrist stability than the flexors.

## Methods

### Participants

Experimental procedures were approved by the research ethics boards (REB) of Brock University (REB# 16-263) and Ontario Tech University (REB# 15044). Written consent was obtained from all participants prior to the experiment. Twelve right-handed males (Height: 180.2 ± 7.7 cm; Weight: 77.4 ± 10.4 kg; Age: 23.9 ± 2.7 years) were recruited for this study. Participants were excluded if they presented with any upper-body, neuromuscular injuries.

### Experimental Setup

**Figure 1:**
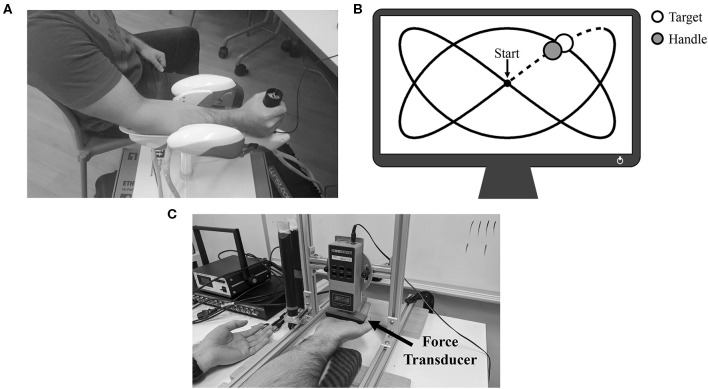
**(A)** Experimental setup for the tracking trials. Participant's forearm is positioned atop the WristBot support, and their hand is gripping the handle of the device. **(B)** Example of the 2:3 Lissajous curve. The white circle represents the target as it moves around the curve, while the gray circle represents the real-time position of the handle. Data collected during the initial dotted-line portion was not analyzed. **(C)** Experimental setup for MVCs and the sustained isometric fatigue trial. In this example, the participant was setup for wrist flexion MVCs/wrist flexion fatigue.

Participants were seated with their dominant forearm supported in a three-degrees-of-freedom wrist manipulandum (WristBot, Genoa, Italy; Masia et al., 2009; Iandolo et al., 2019) with their hand firmly gripping the device's handle (Figure 1A). All participants had previous experience using the manipulandum (Forman et al., 2020). The manipulandum was positioned at a comfortable distance so that subjects neither leaned forwards nor sideways. While upper-limb position was not controlled between participants, upper-limb joint angles *were* manually assessed using a goniometer and matched between the two experimental sessions (elbow extension: 134.0 ± 3.4°; shoulder flexion: 33.0 ± 7.4°; shoulder external rotation: 36.0 ± 4.2°). As a group, participants' grasp distance (distance from the wrist crease to the middle of the manipulandum's handle) was 7.9 ± 0.5 cm. The position of the handle was digitally displayed to participants on a computer monitor as a blue circle that could be moved horizontally (wrist flexion/extension) and vertically (radial/ulnar deviation of the wrist) by moving the handle of the WristBot. For all tracking trials, participants were instructed to overlay their blue circle (by moving the device's handle) on the monitor with a yellow target circle that moved along a set path. This path was a 2:3 Lissajous curve that was ±32° in the x-axis (flexion-extension) and ±18° in the y-axis (radial-ulnar deviation). The circular yellow target took 20 s to complete one full cycle of the Lissajous curve (see Figure 1B for an example of the monitor display). Thus, movement velocity was controlled by the target and consistent across trials. A single lap of the Lissajous curve represented a single tracking trial. The tracking trials were non-fatiguing as no resistance was provided to participants from the manipulandum. Trials were performed both prior to and following a single bout of maximal sustained isometric contraction.

### Experimental Protocol

**Figure 2:**
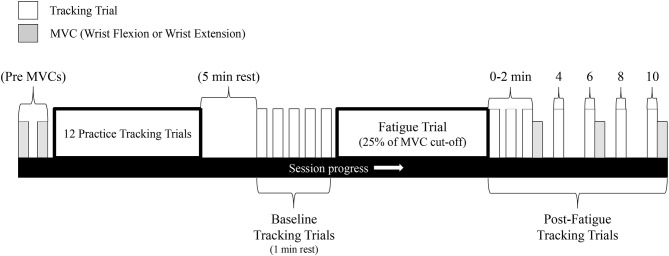
Schematic of the experimental protocol. This protocol was repeated for both the wrist flexion fatigue session and the wrist extension fatigue session (sessions separated by 7 days). Gray bars represent MVCs while white bars represent a single tracking completion of the Lissajous curve.

This experiment consisted of two separate testing sessions. Each session was separated by 7 days and consisted of either (1) maximal sustained isometric wrist flexion, or (2) maximal sustained isometric wrist extension (order was pseudorandomized across sample; 6 participants started with flexion, 6 started with extension). A visual overview of the experimental protocol can be seen in Figure 2.

Upon obtaining informed consent, participants were seated in front of a table-mounted force transducer (Model: BG 500, Mark-10 Corporation, New York, USA). In this same seat, the WristBot rested at the participant's right side. The transducer was raised above the table to allow participants to place their right hand underneath (Figure 1C). For the wrist flexion session, the transducer made contact with the distal anterior surface of the metacarpal bones (top of the palm), while for the wrist extension session, the transducer made contact with the distal posterior surface of the metacarpal bones (back of the most proximal knuckles). This placement for both sessions was marked on the hand with a black marker to match alignment throughout the experiment. For both sessions, the angle of the wrist was maintained at neutral (neither flexed nor extended). Participants then performed two maximal voluntary contractions (MVCs) held for 3–4 s and separated by 1 min of rest. For the MVCs, participants were required to maximally flex/extend (flex on flexion fatigue day/extend on extension fatigue day) their wrist upwards against the force transducer with their right hand open (phalanges extended). Participants were instructed to maintain an open hand throughout the MVC and to keep their forearm firmly upon the support pads (to isolate wrist forces and limit any assistance from the elbow flexors). Their left hand was placed on the table beside them, supinated and palm open so as not to provide them with any additional assistance. Participants were provided with ample verbal encouragement from the researchers for both MVCs as well as visual feedback of their MVC force. The greater of the two trials was deemed their true MVC.

While remaining in the same seat, participants placed their right hand in the WristBot's support and grasped the handle of the manipulandum. Relevant joint angles of the upper-limb (see *Experimental setup* above) were assessed at this time. Due to the novelty of the tracking task, it was vitally important to limit the influence of motor learning on performance outcomes in the present study. To accomplish this, participants first performed 12 practice tracking trials with each trial separated by 1 min of rest. **[**12 trials were deemed sufficient based on preliminary pilot work. In these pilot sessions, 5 participants performed 20 trials of the Lissajous curves with 1 min of rest between trials. Mean tracking error (see *Data analysis* for explanation) rapidly decreased after the first two trials but only gradually improved after trial 3. Group tracking error did not significantly improve after trial 12**]**. Following the 12 practice trials, participants were given 5 min of rest. Baseline (pre-fatigue) tracking trials were then performed with a total of 5 trials separated by 1 min of rest each.

For the fatigue-inducing trial, participants placed their right hand back underneath the table-mounted force transducer. Fatigue was then induced by a maximal sustained isometric wrist flexion/extension (on separate days) MVC. The MVC was performed following the same guidelines as mentioned above (hand open on both days). The cut-off criteria for the sustained MVC was when participants could no longer maintain 25% of their pre-fatigue MVC force. This cut-off criteria was not disclosed to participants, who were instead told that they would be exerting maximal force for ~1–2 min. They were to relax only once the researchers (who were actively watching the force readings) told them to stop. Ample verbal encouragement was provided to participants throughout the fatigue-inducing trial.

Following the 25% cut-off, participants immediately returned their right hand to the WristBot and performed their first post-fatigue tracking trial. While the time between the end of the fatigue-inducing trial and the start of the first post-fatigue tracking trial was not measured, it is estimated that it took ~5 s to get participants back into the WristBot and begin tracking. The first tracking trial was labeled as “0” min post-fatigue. Additional tracking trials also occurred at 1, 2, 4, 6, 8, and 10 min post-fatigue. An MVC was performed immediately after the tracking trials at 2, 6, and 10 min post-fatigue to assess wrist flexion/extension force recovery (flexion MVCs on flexion fatigue day/extension MVCs on extension fatigue day). These MVCs were not sustained, and only lasted ~3–4 s.

### Data Analysis

Kinematic data of both the manipulandum's handle (which represents the participant's hand position) and the monitor-displayed target were sampled at 100 Hz and analyzed off-line (Matlab 2015b, Mathworks Inc., Natick, MA, USA). Because participants had to “catch-up” to the target once each tracking trial began, the initial portion of the Lissajous curve (right before the first turn; the dotted section in Figure 1B) was not assessed in this study. A 6th order Savitzky-Golay filter was used to smooth the positional data in the x and y-axis (Squeri et al., 2010). The Savitzky-Golay is a polynomial fitting filter that works through the means of linear least squares. This filter is segmented in that it fits separate polynomials to a subset of data points within a predetermined window length. The window length in the present study was set to 170 ms which functions as an equivalent 11 Hz low-pass filter (Squeri et al., 2010). From this data, two groups of metrics were used to quantify performance. These include: (1) tracking error and various subtypes, and (2) movement smoothness.

**Tracking Error:** Was calculated as the square root of the displacement between the position of the handle and the position of the target and is calculated by the following formula:

$$\begin{array}{l} |\overset{⇀}{e}|=\;\sqrt{{\left(H_{x}-T_{x}\right)}^{2}+{\left(H_{y}-T_{y}\right)}^{2}} \end{array}$$

where $\left|\overset{⇀}{e}\right|$ is the Euclidean distance, and *H* and *T* are the positions (x, y coordinates) of the handle and target, respectively. The error at each data point was summed over the full tracking trial and divided by the total number of samples to give mean tracking error. To provide additional insight into possible error patterns, we also separated tracking error into the 4 main movement directions: left, right, up, and down on the computer monitor, which was made possible primarily by flexion of the wrist, extension of the wrist, radial deviation, and ulnar deviation, respectively.

**Longitudinal Component:** Is a measure of whether the handle is ahead of or behind the target at each data point. If the resulting value is positive, then the handle is ahead of the target relative to the target's own trajectory, and is given by the following formula:

$$\begin{array}{l} \overset{⇀}{u_{l}}=\frac{1}{\sqrt{{{\dot{T}}_{x}}^{2}+{{\dot{T}}_{y}}^{2}}}\;\left[\begin{array}{c} {\dot{T}}_{x}\\ {\dot{T}}_{y} \end{array}\right]=\;\left[\begin{array}{c} ux_{l}\\ uy_{l} \end{array}\right]\\ \delta_{l}=\;\overset{⇀}{e}\;\cdot \;\overset{⇀}{u_{l}} \end{array}$$

where $\overset{⇀}{u_{l}}$ is the unit vector of the trajectory of the target at each data point, and δ_*l*_ is the longitudinal component of the tracking error. In order to establish whether the handle is ahead of or behind the target, the direction of the trajectory of the target must first be established. The first derivative is taken of the target displacement to find the tangent vector to the trajectory. The norm of the obtained vector is calculated. Then the tangent vector is normalized to obtain the unit vector. $\overset{⇀}{u_{l}}$ is then multiplied against the error vector measured from earlier ($\overset{⇀}{e}$, see Tracking Error) which gives either a positive (handle is ahead of the target) or negative (handle is behind the target) value.

**Normal Component:** Is a measure of whether the handle is to the right or left of the target at each data point. If the resulting value is positive, then the handle is to the right of the target relative to the target's own trajectory, and is given by the following formula:

$$\begin{array}{l} \overset{⇀}{u_{n}}=\left[\begin{array}{c} uy_{l}\\ {-ux}_{l} \end{array}\right]\\ \delta_{n}=\;\overset{⇀}{e}\;\cdot \;\overset{⇀}{u_{n}} \end{array}$$

like the longitudinal component, in order to establish whether the handle is to the right or left of the target, the direction of trajectory of the target must first be established. This is given by the $\overset{⇀}{u_{l}}$ equation described earlier. $\overset{⇀}{u_{n}}$ is simply the orthogonal of $\overset{⇀}{u_{l}}$ and is then multiplied by the error vector. This then gives either a positive (handle is to the right of the target) or negative (handle is to the left of the target) value.

**Figural Error:** Is a measure of how accurately the participant's trajectory adheres to the ideal target trajectory (or how well the participant recreates the target path/shape; Conditt et al., 1997). This measure is insensitive to speed, meaning that it does not matter if an individual is ahead of or behind the target. The measure is given by the following equations:

$$\begin{array}{l} dist_{AB}\left(i\right)=\;\underset{j}{\text{min}}\|A_{i}-B_{j}\|\;i=1,\;2,\;\ldots n\\ dist_{BA}\left(j\right)=\;\underset{i}{\text{min}}\|A_{i}-B_{j}\|\;j=1,\;2,\;\ldots m\\ \;FE_{AB}=\;\frac{\sum_{i=1}^{n}dist_{AB}\left(i\right)+\;\sum_{j=1}^{m}dist_{BA}\left(j\right)}{n+m} \end{array}$$

where “*A*” and “*m*” are the time series and total samples of the target trajectory and “*B*” and “*n*” are the time series and total samples of the handle trajectory. The first equation calculates the distance between a single data point of the target (denoted by *j*) and every data point of the handle (in the present study, 2,000 samples) before moving to the next target data point. The minimum distance of all these comparisons is then taken. **[**For example, if at sample number 100, the position of the handle was directly overlaying some portion of the Lissajous curve (this could be at any point along the target trajectory) the minimum distance would be zero**]**. The second equation is the same, but in reverse, and compares every data point of the target against a single data point of the handle. The final equation adds the sum of all the minimum distances and divides it by the sum of the two samples. A final figural error score of 0 would indicate that the handle was directly overlaying the target trajectory throughout the entire trial.

**Jerk Ratio:** Following filtering with the 6th order Savitzky-Golay filter, the displacement data of both the handle and the target were taken to the 3rd differential in order to obtain jerk. The jerk ratio was calculated as the integrated squared jerk (ISJ) (Platz et al., 1994) of the handle divided by the ideal ISJ of the target. ISJ was defined as $\int \left({\dddot{H}}_{x}^{2}+{\dddot{H}}_{y}^{2}\right)dt$ and integrated over the entire tracking trial. As the jerk ratio in the present study is a comparison of the handle to the target, a value of 1 would represent movement that is as smooth as possible. Any value greater than 1 signifies movement that is less smooth than the movement of the target.

### Statistics

Statistical analyses were performed using SPSS software (SPSS, IBM Corporation, Armonk, NY, USA). Assumptions of sphericity were tested with Mauchley's test of sphericity, and in cases where violated, degrees of freedom were corrected with Greenhouse-Geisser. A two-way repeated measures ANOVA (fatigue day x measurement time) was conducted for MVC data, all tracking error metrics, and jerk ratio measures to identify differences between both the two fatigue sessions as well as between baseline and following sustained isometric fatigue. In cases where a main effect of measurement time was found, *post-hoc* pairwise comparisons were conducted with a Bonferroni correction. Effect sizes (ES) were evaluated using partial ETA squared calculated as the division of the sum of squares of the effects (SS_Effect_) by both the SS_Effect_ and the sum of squares of the error (SS_Error_). Significance level was set at *P* < 0.05. Statistical analyses were performed on kinematic data in degrees (°). However, to more clearly communicate experimental findings, data has been shown in some figures as normalized to baseline measures. Group data is reported as mean ± SD.

## Results

### MVC Force

**Figure 3:**
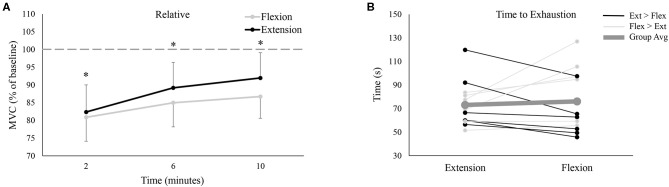
**(A)** Group averages of relative (% of baseline) MVC force between the wrist flexion and wrist extension sessions. Gray lines depict wrist flexion MVC force collected on the wrist flexion fatigue day, while black lines represent wrist extension MVC force collected on the wrist extension fatigue day. The x-axis denotes the time of collection; the numbers refer to time of collection after fatigue. The dotted-line represents pre-fatigue (or baseline) MVC force. * denotes a significant difference of MVC force at one time point to all other time points. **(B)** Time to exhaustion for all 12 participants on both testing sessions. Black lines represent the 6 participants who took longer to fatigue on the extension day; light gray lines denote the 6 participants who took longer to fatigue on the flexion day. Lastly, the thick, dark gray line shows the group average.

Participants produced significantly more absolute force in wrist flexion than wrist extension during the pre-fatigue MVCs (Flexion: 176.1 ± 38.2 N, Extension: 157.6 ± 34.6 N, *P* < 0.05). Absolute MVC force significantly decreased following the fatigue-inducing trial, regardless of testing session, and remained significantly reduced from baseline all the way to 10-min post-fatigue (*P* < 0.05 for all three post-fatigue time points). Normalized to baseline pre-fatigue MVC force, Figure 3A shows relative MVC force in the post-fatigue recovery period. In this recovery period, there were no significant differences between the flexion or extension sessions [*F*_(3, 33)_ = 3.084, *P* = 0.11, ES = 0.22], nor was there an interaction effect of session and time [*F*_(3, 33)_ = 2.25, *P* = 0.13, ES = 0.17]. However, there was a main effect of time [*F*_(3, 33)_ = 53.86, *P* < 0.05, ES = 0.83] with relative MVC forces different from each other at all time points (*P* < 0.05 for all comparisons). This indicates that at each subsequent time point, MVC force was significantly recovering from the previous time point.

### Time to Exhaustion

Group data (Figure 3B, dashed line) demonstrated that there was no difference in the time it took for participants to reach their 25% of pre-MVC force cut-off (Flexion: 76.1 ± 26.8 s, Extension: 73.1 ± 19.3 s, *P* = 0.65). However, there was tremendous variability across participants, with 6 participants taking longer to fatigue during extension (black lines of Figure 3B) and 6 participants taking longer to fatigue during flexion (light gray lines of Figure 3B).

### Tracking Error

**Figure 4:**
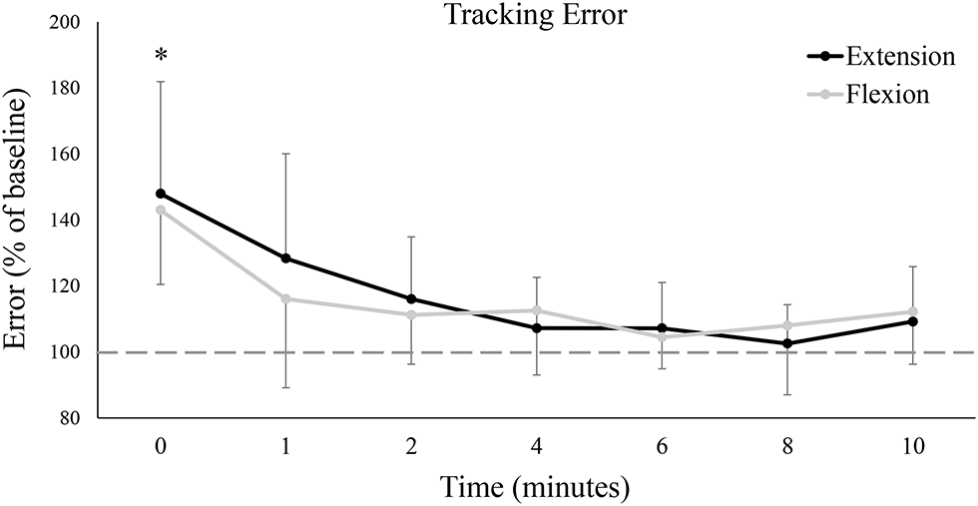
Group averages of mean tracking error calculated over the entire Lissajous curve (excluding the dotted-line portion). Tracking error is normalized to baseline (shown by the dashed horizontal line), and data points are shown in minutes after fatigue (0–10). Black lines represent tracking error from the wrist extension fatigue session, while gray lines represent tracking error from the wrist flexion fatigue session. * denotes a significant difference of both extension and flexion compared to baseline.

**Figure 5:**
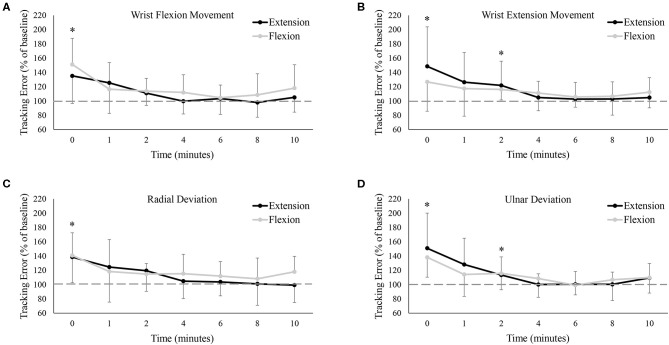
Group averages of mean tracking error **(A)** only when the wrist was flexing, **(B)** only when the wrist was extending, **(C)** only when the wrist was moving in radial deviation, and **(D)** only when the wrist was moving in ulnar deviation. Tracking error is normalized to baseline (shown by the dashed horizontal lines in each graph), and data points are shown in minutes after fatigue (0–10). Black lines represent tracking error from the wrist extension fatigue session, while gray lines represent tracking error from the wrist flexion fatigue session. * denotes a significant difference of both extension and flexion compared to baseline.

Figure 4 shows group data for mean tracking error calculated over the full Lissajous curve. While statistical analyses found no difference between the extension and flexion sessions [*F*_(7, 77)_ = 0.06, *P* = 0.81, ES = 0.01], there was a main effect of time on tracking error [*F*_(7, 77)_ = 12.35, *P* < 0.05, ES = 0.53], with error significantly worse from baseline immediately post-fatigue (Baseline: 1.40 ± 0.54°, 0: 2.02 ± 0.51°, *P* < 0.05). Although group means never returned (or fell below) baseline error (even up to 10 min post-fatigue), tracking error recovered rapidly and was not significantly different from baseline at, or following, 1 min post-fatigue. To examine if there were any movement-specific trends, tracking error was separated into the four primary movement directions (Figure 5). However, results were mostly similar to data calculated over the full curve. There was no difference in tracking error between the extension and flexion sessions for any movement direction, although all four directions showed a main effect of time [Flexion movement: *F*_(7, 77)_ = 6.32, *P* < 0.05, ES = 0.37; Extension movement: *F*_(7, 77)_ = 4.04, *P* < 0.05, ES = 0.27; Radial deviation: *F*_(7, 77)_ = 4.42, *P* < 0.05, ES = 0.29; Ulnar deviation: *F*_(7, 77)_ = 8.99, *P* < 0.05, ES = 0.45]. Error significantly increased immediately post-fatigue in both the flexion (Baseline: 1.40 ± 0.71°, 0: 2.02 ± 0.65°, *P* < 0.05) and radial (Baseline: 1.39 ± 0.57°, 0: 1.90 ± 0.56°, *P* < 0.05) directions, but was not significantly different from baseline at, or following, 1-min post-fatigue. Error was also significantly greater immediately post-fatigue during extension (Baseline: 1.36 ± 0.48°, 0: 1.80 ± 0.56°, *P* < 0.05) and ulnar (Baseline: 1.40 ± 0.56°, 0: 2.00 ± 0.63°, *P* < 0.05) movement, however, error was also greater at 2-min post-fatigue (Extension: Baseline: 1.36 ± 0.48°, 0: 1.60 ± 0.48°, *P* < 0.05; Ulnar: Baseline: 1.40 ± 0.56°, 0: 1.59 ± 0.43°, *P* < 0.05).

### Longitudinal and Normal Error Components

**Figure 6:**
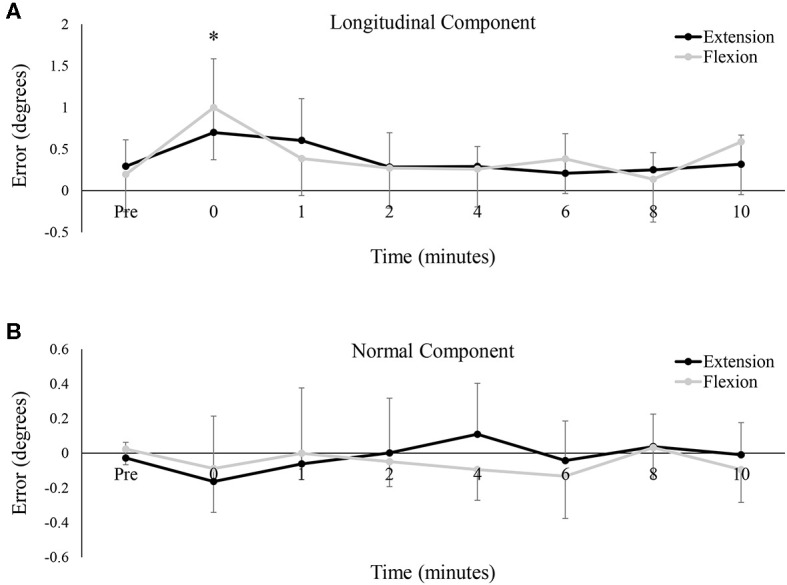
Group averages of the mean **(A)** longitudinal component of the tracking error, and **(B)** the normal component of the tracking error. For both metrics, error is shown in degrees (°) and data points are shown from pre-fatigue to 10 min-post. Black lines represent tracking error from the wrist extension fatigue session, while gray lines represent tracking error from the wrist flexion fatigue session. * denotes a significant difference of both extension and flexion compared to baseline.

Figures 6A,B depict group data of the longitudinal (ahead or behind) and normal (right or left) components, respectively, of the tracking error. As a group, participants tended to rush ahead of the target as it moved around the Lissajous curve. Even at baseline, the longitudinal component averaged 0.24 ± 0.61° between the two testing sessions. This tendency increased immediately post-fatigue, with participants significantly farther ahead than at baseline (Baseline: 0.24 ± 0.61°, 0: 0.85 ± 0.70°, *P* < 0.05). However, there was no difference in the longitudinal component between testing sessions, and the error across both sessions was not significantly different from baseline at, or following, 1-min post-fatigue. Regarding the normal component of error, data seemed to hover around 0 for all conditions, meaning that as a group, participants missed the target to the right and to left to a nearly equal extent. The normal component of error was not significantly different between the two testing sessions [*F*_(7, 77)_ = 0.52, *P* = 0.50, ES = 0.05], nor did it significantly change over time [*F*_(7, 77)_ = 1.61, *P* = 0.21, ES = 0.13].

### Figural Error and Jerk Ratio

**Figure 7:**
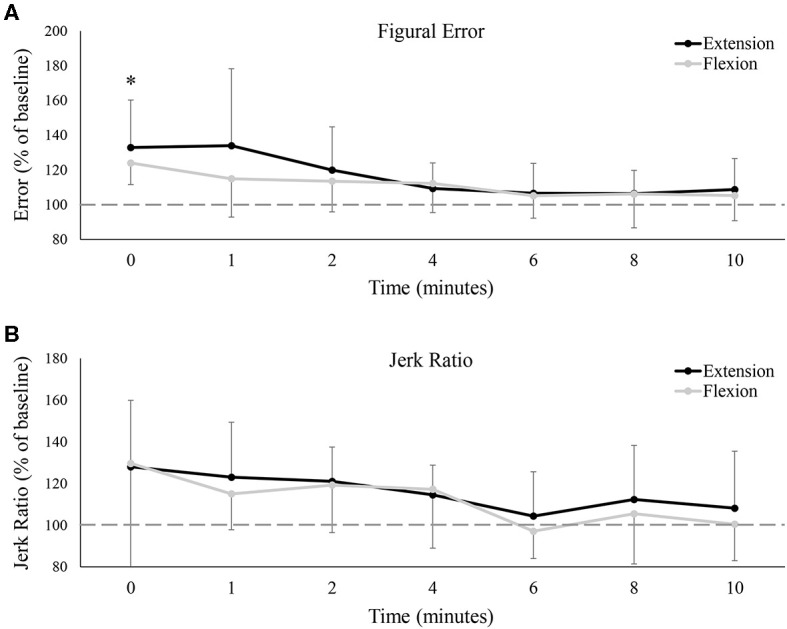
**(A)** Group averages of figural error, and **(B)** group averages of the jerk ratio. Error is relative to baseline (shown by the dashed horizontal lines in each graph), and data points are shown in minutes after fatigue (0–10). Black lines represent tracking error from the wrist extension fatigue session, while gray lines represent tracking error from the wrist flexion fatigue session. * denotes a significant difference of both extension and flexion compared to baseline.

Group data on figural error is shown in Figure 7A, and much like tracking error, demonstrated a main effect of time [*F*_(7, 77)_ = 7.10, *P* < 0.05, ES = 0.392], with no difference between the flexion and extension sessions [*F*_(7, 77)_ = 1.55, *P* = 0.24, ES = 0.12]. Figural error significantly increased immediately post-fatigue (Baseline: 0.74 ± 0.19°, 0: 0.94 ± 0.22°, *P* < 0.05), but was not significantly different from baseline at, or following, 1-min. Jerk ratios (representing movement smoothness) also demonstrated a main effect of time [*F*_(7, 77)_ = 3.37, *P* < 0.05, ES = 0.23], although interestingly, pairwise comparisons revealed no differences between any two time points. There was also no difference between the flexion and extension test sessions on jerk ratios [*F*_(7, 77)_ = 0.07, *P* = 0.79, ES = 0.01].

## Discussion

To the best of our knowledge, this is the first study to have examined sustained isometric MVCs in opposing forearm muscle groups and their subsequent influence on precision movements of the hand and wrist. Results demonstrated that hand-tracking accuracy was impaired across nearly all analysis metrics, although most of these measures recovered within 1-min. Increased tracking error seemed to be mostly consistent throughout the full trace; minimal differences were seen when examined in the four primary directions. A tendency to rush (longitudinal component) while tracking may explain part of the increased tracking error; left/right error tendencies averaged out to be the same following fatigue. However, the most interesting finding of this study may be the lack of differences between the flexion and extension fatigue sessions. Across all metrics, there were no differences in tracking proficiency whether the wrist flexors or the wrist extensors were fatigued. This raises new questions regarding the functional roles of the wrist flexors and extensors in both sustained contractions and in the execution of fine motor skills.

### Flexors vs. Extensors

Prior to the fatigue-inducing trial, participants produced significantly more absolute wrist flexion MVC force than wrist extension, although when normalized (Figure 3A), there were no differences in MVC force recovery. For every other metric, there were also no differences between the two sessions. Participants produced equal tracking error between the wrist flexion and extension sessions and even demonstrated similar error tendencies (Figures 6, 7). This finding was surprising, given the differences in anatomy and physiological roles of the two muscle groups. The wrist flexors possess a physiological cross-section area (PCSA) that is approximately twice as large as the wrist extensors (Flexors: ~24.8 cm^2^, Extensors: ~11.8 cm^2^; Lieber et al., 1990, 1992; Jacobson et al., 1992). The wrist flexors also cumulatively possess larger moment arms than the wrist extensors (Gonzalez et al., 1997). Since PCSA is a strong predictor of a muscle's strength, these two factors indicate that the wrist flexors are capable of generating a peak moment in excess of 2:1 of the extensors (Gonzalez et al., 1997). Muscle lines of action, at least in the most superficial forearm muscles, only compound this force-generating disparity. Both the flexor carpi radialis (FCR) and ulnaris (FCU) possess a mostly direct line of action toward wrist flexion (Bawa et al., 2000). In contrast, the extensor carpi radialis (ECR) and ulnaris (ECU) are more closely aligned with radial and ulnar deviation, respectively (Bawa et al., 2000). Consequently, the wrist extensors must function at a higher percentage of maximal activation in order to counterbalance the activity of the stronger flexors, particularly in movements that are closer to the flexors' more direct line of action. This has been repeatedly shown experimentally, whereby the wrist extensors exhibit significantly higher levels of muscle activity than the flexors across a multitude of handgrip and wrist forces (Snijders et al., 1987; Mogk and Keir, 2003; Forman et al., 2019). These recruitment characteristics have contributed to the notion that the wrist extensors make a greater contribution to wrist joint stability (Holmes et al., 2015). They are also the primary reason why the wrist extensors exhibit an earlier onset of fatigue than the wrist flexors (Hägg and Milerad, 1997). Considering all of the above, it was well within reason to hypothesize that separately inducing fatigue through wrist flexion and wrist extension would result in unique performance impairments.

The most plausible explanation for this lack of difference may arise from the recruitment characteristics of the wrist extensors. In a recent study (Forman et al., 2019), we demonstrated that during isolated wrist extension trials, the wrist flexors averaged just 2.8% of maximal activity (vs. 23.2% for the extensors). However, in the pure wrist flexion trials, the wrist extensors averaged 9.8% of maximal activity (compared to 28.1% in the flexors). To summarize, the wrist extensors were highly active even in pure wrist flexion exertions, while the opposite was not true. Thus, in the present study, it is entirely possible that prolonged wrist extension successfully isolated the wrist extensor muscles (i.e., the wrist flexor muscles were not fatigued). However, during prolonged wrist flexor fatigue, literature would suggest that both wrist flexors and extensors may have fatigued simultaneously (Forman et al., 2019). If so, then the wrist flexors were only fatigued in a single session of the present study, while the wrist extensors were fatigued in both. The two sessions would therefore be more similar than previously assumed. However, this also further complicates the comparison between the two muscle groups in regards to fatigue; it would be very difficult to isolate the two using any form of exercise. Future investigations examining antagonist fatigue metrics following agonist fatigue (i.e., is wrist extension MVC impaired following prolonged wrist flexion, and vice versa?), or using alternative fatigue measures (such as surface electromyography), would help address these questions.

Finally, it is possible that during both the flexion and extension fatigue-inducing sessions, compensation from synergistic muscles diminished the influence of fatigue on tracking accuracy measures. For instance, in studies that have induced isolated fatigue in single muscles (i.e., vastus lateralis), the muscle activity of adjacent/synergistic muscles increases during synchronous muscles actions (i.e., knee extension) (Akima et al., 2002; Stutzig et al., 2012; Stutzig and Siebert, 2015a,b). Thus, while fatigue of the wrist flexors or extensors might be uniquely detrimental to tracking accuracy of the hand/wrist in isolation, compensation from synergistic muscles may diminish these differences during the execution of complex motor tasks. It is unclear if this was the case in the present study, given that the fatigue-inducing task likely fatigued forearm muscles on a “global” scale (the prime movers and synergists might have fatigued simultaneously). Regardless, this suggestion requires further investigation.

### Time to Exhaustion

To fairly compare tracking error between fatigue-inducing contractions of the wrist flexors and extensors, a relative cut-off criterion (25% of MVC for each session) was established. Interestingly, although there was large variability between participants, the duration of the fatigue-inducing trial averaged across our sample was ultimately not different between days; the wrist flexors took just as long to reach the 25% cut-off as the extensors. This is surprising given the differences in function and muscle architecture of the two muscle groups. Direct assessments of joint stiffness (Holmes et al., 2015) and investigations using muscle activity and co-contraction as surrogate measures for joint stiffness (Hägg and Milerad, 1997; Mogk and Keir, 2003; Forman et al., 2019) suggest that the wrist extensors contribute more to wrist stability than the flexors. While we are not aware of any study that has directly quantified muscle fiber typing between the wrist flexors/extensors of the forearm (fiber typing research in the forearm is overall scarce; Fugl-Meyer et al., 1982; McIntosh et al., 1985; Mizuno et al., 1994), stabilizing muscles of the trunk are predominantly composed of type 1 fibers (Mannion et al., 1997; Boyd-Clark et al., 2001; Arbanas et al., 2009). Thus, considering the stabilizing behavior of the wrist extensors, and the possibility that they possess a greater ratio of type 1 muscle fibers (at this time, this point is pure speculation), it was hypothesized that the flexors would fatigue faster than the extensors. Given our experimental setup (forearm supinated for flexion fatigue; pronated for extension), it is possible that forearm posture contributed to this lack of difference (La Delfa et al., 2015; Yoshii et al., 2015). However, while the average time to exhaustion was not different between sessions, there was large variability between participants; 6 participants took longer to fatigue during flexion; 6 took longer to fatigue during extension. It is possible that certain anthropometric characteristics, such as hand size, hand length, or forearm muscle moment arms predisposed some individuals to fatiguing earlier in one session over the other. Additionally, the participants recruited in the present study were highly active (varsity athletes and strength athletes). Differences in training status, or differences in inter-flexor/extensor strength, may have also contributed to a certain fatigue predisposition.

### Performance Fatigability and Hand Tracking

In the present study, isometric fatigue significantly impaired hand-tracking performance immediately after the cessation of the fatigue trial. Tracking deficits mostly recovered within 1-min post-fatigue, although tracking in certain directions (wrist extension and ulnar deviation) remained impaired for up to 2 min. A shift in error tendencies was also observed; participants were more inclined to be ahead of the target immediately following fatigue as compared to baseline. While there was no *net change* in left/right error tendencies throughout the study, Figure 7A suggests that participants were tracking with greater *absolute* left/right error following fatigue. The equation used to calculate figural error is insensitive to time, and thus, any increase in figural error must be due to greater deviations to the left/right of the target pathway.

This is not the first study to report fatigue-induced accuracy impairments. Indeed, these findings are well-supported by literature (Jaric et al., 1999; Huysmans et al., 2008; Missenard et al., 2008b). Although separate laboratory groups have attributed fatigue-induced accuracy deficits to isolated mechanisms, the underlying cause is almost certainly multi-factorial. Literature has reported numerous factors, including decreased force availability (Jones et al., 2002), greater signal-dependent noise (Missenard et al., 2008a), slower contractile speed (de Haan et al., 1989), decreased co-contraction during precision movements (Gribble et al., 2003; Missenard et al., 2008b), and impaired proprioception (Pedersen et al., 1999; Mugnosso et al., 2019). Force availability is likely linked with signal-dependent noise (SDN), where “signal” is the optimal force output to execute a given task, and “noise” is any force deviation from that ideal. Should a fatigue-inducing task decrease available force (i.e., decrease MVC), a larger relative portion of available force is then required to complete tasks with absolute force requirements (the force required to move the handle in the present study). This is noteworthy as SDN (force variability) increases linearly with higher force outputs (Jones et al., 2002) and is greater still following fatigue (Missenard et al., 2008a). Greater fatigue-induced force variability would have assuredly contributed to tracking error in the present study where accurate tracking required precise movements and speed. Speed itself may help explain why participants tended to rush the target (Figure 6A). Both maximal velocity and maximal torque decrease following fatigue (Buttelli et al., 1996); muscle relaxation-time is similarly prolonged (de Haan et al., 1989). In precision tasks, this has manifested experimentally as a reduction in peak velocity when rapidly moving to a known target (Jaric et al., 1997). In the present study, it is possible that participants altered their tracking strategy following fatigue. To compensate for a potential loss of available speed, participants may have opted to remain ahead of the target (even at the expense of error) so as to avoid the poorer alternative of falling behind and struggling to catch-up.

While neither co-contraction nor proprioception were assessed in this study, both factors have been reported as significant contributors to movement accuracy. Greater co-contraction increases limb impedance (Osu and Gomi, 1999), reduces kinematic variability (Selen et al., 2005), and subsequently leads to improved accuracy (Gribble et al., 2003). This is all relevant given that performance fatigability impairs co-contraction to a similar extent as tracking error, even when force availability is controlled for (Missenard et al., 2008b). Finally, extensive evidence has demonstrated that fatigue results in significant joint position sense impairment (Carpenter et al., 1998; Pedersen et al., 1999; Björklund et al., 2003; Roberts et al., 2003). This impairment arises from numerous factors, including, but not limited to, decreased discharge rate of muscle spindles (Macefield et al., 1991), decreased activity of golgi tendon organs (Hutton and Nelson, 1986), and alterations in central pathways (Sharpe and Miles, 1993; Zabihhosseinian et al., 2015). The extent to which co-contraction and proprioception may have influenced the findings of the present study is unclear, and future investigations quantifying these measures at the forearm would add valuable insight.

### Central and Peripheral Mechanisms

In the presence of performance fatigability, changes occur at all levels of the neuromuscular pathway, from the central nervous system (CNS), the neuromuscular junction (NMJ), and the muscle fibers themselves. Research utilizing isometric fatigue-inducing tasks would suggest that these mechanisms likely had some influence over our study's findings. At the level of the motor unit, ample literature has shown that performance fatigability decreases motor unit firing rates (Grimby and Hannerz, 1977; Petrofsky, 1980; Petrofsky and Lind, 1980; Bigland-Ritchie et al., 1983; Woods et al., 1987; Peters and Fuglevand, 1999; Gandevia, 2001). A similar decrease in spinal excitability (Taylor et al., 1996, 2000; Butler et al., 2003; Taylor and Gandevia, 2008) suggests that alpha motoneurones are inhibited, either through intrinsic motoneuron adaptations or peripheral inhibitory pathways (Heckman and Enoka, 2012), in the presence of fatigue. These changes can all occur without a subsequent reduction in force (in non-maximal contractions), which is made possible by a simultaneous increase in motor unit recruitment (Edwards and Lippold, 1956; Scherrer and Bourguignon, 1959; Eason, 1960; DeVries, 1968; Lynn et al., 1978; Johnson et al., 2004; Hendrix et al., 2009). Greater recruitment likely results from an increase in descending neural drive, as studies utilizing transcranial magnetic stimulation (TMS) have shown motor evoked potentials (MEPs) to increase following fatigue (Søgaard et al., 2006; Klass et al., 2008; Lévénez et al., 2008). Increased neural drive (or cortical excitability) is thought to act as a compensatory mechanism to ensure adequate recruitment in the presence of decreased spinal excitability. Interestingly, in these studies, both corticospinal and spinal excitability change rapidly following the cessation of a fatigue-inducing task, either returning to baseline or overcorrecting in ~1-min post-fatigue. This is remarkably similar to the patterns observed in the present study, whereby metrics of tracking error significantly worsened immediately post-fatigue but mostly recovered following 1-min of recovery. It is therefore possible that fatigue-induced changes in central pathways altered voluntary activation in the present study, which may have driven patterns of tracking accuracy post-fatigue.

In terms of peripheral mechanisms, resting twitch force evoked by electrical stimulation of motor point decreases following a sustained MVC (Gandevia et al., 1996). Since some studies have found that the compound muscle action potential (M_wave_) is unchanged following either sustained (Bigland-Ritchie et al., 1986) or intermittent (Neyroud et al., 2014) MVCs, this decrease in force is at least partially explained by changes at the intramuscular level. This may be the result of reduced sarcoplasmic reticulum release/impaired renewal of intracellular calcium and a reduced myofibrillar calcium sensitivity (Westerblad et al., 1991; Fitts, 1994; Glaister, 2005; Allen et al., 2008). Importantly, as voluntary activation typically recovers to near pre-fatigue levels within 30 s of recovery from sustained MVCs (Gandevia et al., 1996; Hunter et al., 2006, 2008; Kennedy et al., 2013, 2015), any lasting impairment in voluntary force production is likely due to intramuscular mechanisms. Thus, in the present study, fatigue-induced changes to intramuscular systems are likely the primary reason why MVC force remained lower from baseline all the way up to 10-min post-fatigue. However, this reduction in available MVC force was insufficient to impair tracking accuracy, at least beyond 1–2 min post-fatigue.

### Practical Implications

Given the results of the present study, one could be mistaken for concluding that the effects of fatigue dissipated after 1–2 min, given that most tracking metrics recovered by 1-min. However, it should be reiterated that MVC force was still significantly reduced from baseline up to 10-min post-fatigue (Figure 3A). Thus, participants *were not* tracking better because they were no longer fatigued; they were tracking better *despite* still being fatigued. Similar work has shown that fatigue may not change kinematic measures, despite fatigue manifesting in surface electromyography (Mugnosso et al., 2019). This is particularly relevant for industries that may rely on performance as an indicator of fatigue, from athletic training and health to occupational settings. If fatigue is only identified once movement precision has noticeably worsened, then fatigue was likely present well-beforehand. Delayed identification of workplace fatigue could have the potential to exacerbate the development of chronic overuse injuries. This, however, should be considered in relation to the sample population examined in this study. Collected from young, active adults, the findings of this study may not be ideally suited for generalization to some workplace settings. In one sense, certain workers performing repetitive tasks could be classified as “industrial athletes,” which might lead to a number of similarities to the present sample. In another sense, since such large performance detriments were observed in a young and active sample, these findings might be more pronounced in an older or more vulnerable working population.

Understanding how performance fatigability manifests in the wrist flexors/extensors is an important first step in addressing the earlier fatigue onset (Hägg and Milerad, 1997) and the higher incidence of injury that has characterized the wrist extensors (Shiri et al., 2006). The findings of the present study seem to indicate that fatigue of either muscle group results in similar accuracy losses, as performance metrics were equally impaired between sessions. However, this may have occurred due to difficulties in isolating the muscles of the forearm, given that the wrist extensors exhibit high muscle activity even as the antagonists (Mogk and Keir, 2003; Forman et al., 2019).

### Study Limitations

In the present study, fatigue was induced via a sustained isometric MVC. Given the known differences of how maximal vs. submaximal contractions influence central (Taylor and Gandevia, 2008) and peripheral (Søgaard et al., 2006; Smith et al., 2007) pathways, the results of the present study should not be generalized to lower intensity fatiguing tasks. Likewise, as this study induced fatigue through an isometric contraction, these findings should not be generalized to fatigue induced by dynamic contractions. The present study also induced fatigue using controlled postures. Not only was the wrist flexion day performed in forearm supination and the wrist extension day performed in forearm pronation (forearm posture may have influenced the development of fatigue), but the wrist angle was also maintained at neutral (0° of flexion/extension). Had the wrist been held at a different angle throughout the sustained MVC, there might have been differences in tracking accuracy (Place et al., 2005; Behrens et al., 2019).

Finally, while the objective of the present study was purely to examine the consequences of performance fatigability on precision hand/wrist movements, our discussion has proposed a number of underlying mechanisms to explain our findings. It should be clarified that none of these mechanisms were quantified, and their contribution to the present results are speculative. Future investigations utilizing techniques such as electromyography (EMG) or TMS following a similar fatigue-inducing task would add valuable insight to this work.

## Conclusion

This report was the first investigation to examine performance fatigability of wrist flexion/extension and its influence on hand tracking accuracy. Contrary to our hypothesis, there were no differences in hand tracking errors between the two testing sessions, which raises questions as to how precisely forearm muscles can be isolated in fatigue-inducing studies. Tracking error was impaired similarly for the two muscle groups immediately following a sustained MVC, but mostly recovered at 1-min post-fatigue. However, MVC force remained lower from baseline for all post-fatigue measures, indicating that participants were capable of accurate tracking in the presence of force deficits.

## Data Availability Statement

The datasets generated for this study are available on request to the corresponding author.

## Ethics Statement

The studies involving human participants were reviewed and approved by Brock University Bioscience Research Ethics Board and the Ontario Tech University Ethics Board. The patients/participants provided their written informed consent to participate in this study.

## Author Contributions

Experimental procedures were developed by DF, GF, MM, JZ, BM, and MH. Data was collected by DF and GF and analyzed by DF, GF, MM, and MH. Manuscript was prepared, edited, and approved by DF, GF, MM, JZ, BM, and MH.

## Conflict of Interest

The authors declare that the research was conducted in the absence of any commercial or financial relationships that could be construed as a potential conflict of interest.
